# Sensory Relevance of Strecker Aldehydes in Wines. Preliminary Studies of Its Removal with Different Type of Resins

**DOI:** 10.3390/foods10081711

**Published:** 2021-07-23

**Authors:** Almudena Marrufo-Curtido, Arancha de-la-Fuente-Blanco, María-Pilar Sáenz-Navajas, Vicente Ferreira, Mónica Bueno, Ana Escudero

**Affiliations:** 1 Laboratorio de Análisis del Aroma y Enología (LAAE), Department of Analytical Chemistry, Universidad de Zaragoza, Instituto Agroalimentario de Aragón (IA2) (UNIZAR-CITA), Associate Unit to Instituto de las Ciencias de la Vid y del Vino (ICVV) (UR-CSIC-GR), c/Pedro Cerbuna 12, 50009 Zaragoza, Spain; amarrufo@unizar.es (A.M.-C.); arandlfb@unizar.es (A.d.-l.-F.-B.); vferre@unizar.es (V.F.); mobueno@unizar.es (M.B.); 2 Department of Enology, Instituto de Ciencias de la Vid y del Vino (CSIC-GR-UR), Finca La Grajera, Ctra. de Burgos Km. 6 (LO-20—Salida 13), 26007 Logroño, La Rioja, Spain; mpsaenz@icvv.es

**Keywords:** strecker aldehydes, oxidation, acetaldehyde, resins, orthonasal quality, aldehyde removal

## Abstract

The orthonasal quality of two synthetic contexts of wine (young wine and oaked wine) spiked with six different levels of the Strecker aldehydes (isobutyraldehyde, 2-methylbutanal, 3-methylbutanal, methional and phenylacetaldehyde) was evaluated by a panel of wine experts. The aldehyde levels simulated the concentrations present in wines protected from oxidation during production and storage and after severe oxidation. Significant quality detriments were observed at concentrations of 13 µg/L of methional, 49 µg/L of phenylacetaldehyde, 17 µg/L of isobutyraldehyde, 12 µg/L of 2-methylbutanal and 24 µg/L of 3-methylbutanal. The presence of these levels of aldehyde concentrations induced the reduction of fruitiness in young wines and of woody notes in oaked wines as well as the appearance of the typical attributes that define wine oxidation. More than 75% of recently opened commercial wines contain total levels of Strecker aldehydes higher than those, however their effect is not always noticeable as they are forming inodorous adducts with SO_2_. Nevertheless, this content is a potential risk for the shelf life of the wine, as once SO_2_ is depleted, these aldehydes could release back into their odour-active forms. Thus, in order to reduce the presence of Strecker aldehydes, eight different resins were studied (two scavengers, four mixed-mode anion exchange and two pure anion exchange) in white wine at two levels of SO_2_. After 24-h contact, the mixed mode Strata X-A resin was able to significantly reduce aldehydes’ percentages: between 11% for isobutyraldehyde and 86% for phenylacetaldehyde. On the other hand, wine colour was affected and therefore the applicability of the treatment should be further studied. However, this work can be considered a starting point to solve the technological challenge involved in the elimination of aldehydes from wine.

## 1. Introduction

During bottle ageing, wines are in contact with oxygen to a greater or lesser extent. However, not all wines endure this ageing and oxidise. The clearest symptoms of oxidation are the evolution of wine colour towards yellow and brown hues [1] and the appearance of the oxidised aroma [2]. Usually, the latter happens before wine browning becomes obvious [3,4], even leading to wine spoilage. In fact, 48% of wines identified as faulty in several oenological contexts have defects related to inadequate ageing, in particular with aroma-related oxidation problems [5]. Therefore, it is vital to control wine aroma evolution during ageing to avoid important economic losses and image damage.

The major compounds responsible for oxidation aroma are acetaldehyde and Strecker aldehydes, mainly methional and phenylacetaldehyde [2,6,7]. However, a high proportion (70–95%) of these compounds are present in wine in the form of odourless hydroxyalkylsulfonates [8]. On one hand, the acetaldehyde-SO_2_ adduct can be considered irreversible [9], on the other hand, the SO_2_-adducts formed with Strecker aldehydes have been shown to be reversible [10]. The free forms are released from the non-volatile adducts as the SO_2_ in the wine disappears, either by oxidation or by reaction with other species. In this way, Strecker aldehydes release due to equilibria shifts and thus could be sensory perceived. It has already been reported that the accumulation of aldehydes could be detrimental to wine quality [11], but to date there is no sensory study that determines the concentration of Strecker aldehydes able to decrease the aromatic quality of wines.

Taking into account the relevance of aldehydes and their reactivity, their removal might look interesting in wine as it is necessary in many areas such as pharmacy [12], industry [13], food [14] or biotechnology [15]. Such removal can be carried out in following different approaches such as distillation, gas stripping, pervaporation, solvent extraction, solid phase extraction [16], derivatisation [17] or precipitation and filtration from an organic solvent [15]. Nevertheless, for wine and with the intention of scaling the process of elimination of aldehydes to the winemaking process, it seems that the most appropriate methodology would be to use solid phase extraction with resins. Furthermore, if it were a reversible binding process it would allow the regeneration of the resins, as occurs with the Amberlite resin in the bisulphite form (IRA-SO_3_ H) for the removal of 3-hydroxypropionaldehyde during the biotechnological conversion of glycerol [15].

Up to now, studies for the wine industry have been focused on the effect on organoleptic properties (i.e., colour improvement or haze prevention) of the use of ion exchange resins [18,19,20,21]. Currently the studies in the winery are aimed at the search for treatments that allow for the elimination of substances recognised as wine defects such as acetic acid or ethylphenols. From these ideas, the hypothesis arose of developing a methodology that could eliminate oxidation aldehydes present in wine through reactive polymers endowed with special functional groups [22] or classic-form and modified form ion-exchange resins [23]. Therefore, the objectives of this work are to study about the sensory impact derived from the release of Strecker aldehydes from hydroxyalkylsulfonate adducts during wine storage and to evaluate the potential capacity of different commercial resins to remove acetaldehyde and Strecker aldehydes from wine.

## 2. Materials and Methods

### 2.1. Solvents and Chemicals

Sodium metabisulfite 99% (Na_2_S_2_O_5_), tartaric acid (99%), glycerol (99.5%), 1,2-propanediol (99.5%), sodium hydroxide (98%), ortho phosphoric acid (85%), hydrogen peroxide 3% stabilized *w*/*v* VINIKIT, indicator 4,4, mixed (methyl red-methylene blue) VINIKIT, sodium hydroxide 0.01 mol/L VINIKIT were from Panreac (Barcelona, Spain). Dichloromethane, ethanol and methanol for gas chromatography analyses were purchased from Merk (Darmstadt, Germany). Glyoxal 40% in water were supplied by Sigma–Aldrich (Madrid, Spain). Water was purified in a Milli-Q system from Millipore (Bedford, Germany). Chemical standards were supplied by Sigma-Aldrich, Fluka (Madrid, Spain), ChemService (West Chester, PA, USA) and Firmenich (Switzerland). Their purity is over 90% in all cases, and most of them are over 99%. Specific details can be obtained from method references [10,22,24].

### 2.2. Evaluation of the Sensory Effect of the Release of Strecker Aldehydes from Hydroxyalkylsulfonate Adducts

#### 2.2.1. Wines for Sensory Analysis

Two wine models were generated mimicking: (a) a young wine and (b) an oaked wine. They were prepared by mixing a pool of common non-volatile and volatile wine components, detailed in Table 1, corresponding to the average concentration of aroma compounds found in a previous work [22].

For studying the sensory relevance derived from the release of Strecker aldehydes from hydroxyalkylsulfonate adducts during wine storage for each of the two wine models, five aldehydes (isobutyraldehyde, 2-methylbutanal, 3-methylbutanal, methional and phenylacetaldehyde) were added at six concentration levels based on previous studies [8]. These levels are detailed in Table 2.

These levels were selected in order to simulate the concentration of free aldehydes during different stages of wine ageing. Levels 4, 5 and 6 (6 being the highest level, Table 2) mimic the maximum concentrations of aldehydes that can be released after the breakdown of the hydroxyalkylsulfonates by oxidation processes (in other words, maximal levels of aldehydes in the free forms). Differently, levels 1, 2, and 3 correspond approximately to 10 or 30% (depending on the compound) present in levels 4, 5 and 6. The rationale behind the selection of these two groups of concentration of the free aldehydes is that as stated in the mentioned reference, in a recently opened commercial wine with an adequate SO_2_ level for its preservation, the free forms present in the headspace are 30% of the total amount of isobutyraldehyde and 2-methylbutanal, whereas only the 10% for 3-methylbutanal, methional and phenylacetaldehyde, while the same wine under oxidative conditions, would present most of its aldehydes as free form. Accordingly, these six levels would correspond to three different wines with the same levels of total aldehydes: low category for levels 1 and 4, intermediate category for levels 2 and 5 and high category for levels 3 and 6.

#### 2.2.2. Participants

The sensory tasks were carried out by twenty participants (seven men and thirteen women, ranging from 25 to 63 years old, average = 37 years old) with a long experience in wine aroma evaluation (oenologists and belonging to Laboratory for Flavour Analysis and Enology, LAAE staff) and considered wine experts according to Parr et al. [25].

#### 2.2.3. Procedure

Twenty-mL wine samples were presented in dark ISO-approved wine glasses [26] labelled with 3-digit random codes and covered with plastic petri dishes according to a random arrangement presentation and different for each panellist.

Quality evaluation was carried out in two formal sessions. In the first session, each panellist evaluated six wine samples (one for each spiked level) for young wine samples. In the second session, participants repeated the task but with the six oaked wine samples.

In both sessions, participants were asked to exclusively smell the wine samples orthonasally (without tasting) from left to right. Then, they had to evaluate the perceived quality in a structured 10 cm-scale (anchored with 3 categories: left-end with “low quality”, in the middle “average quality” and right-end “high quality”). The quality score attributed to each wine sample was the distance in cm indicated by each panellist for each wine. Then, to identify the sensory drivers of perceived quality, each participant was requested to freely describe the high and low quality categories by indicating two or three attributes (avoiding hedonic terms) that apply to each quality categories.

#### 2.2.4. Data Analysis

In order to assess how the breakdown of hydroxyalkylsulfonates adducts during wine storage affects the organoleptic quality of the wines, a two-way ANOVA (panellists as random and samples as fix factor) was performed with the panellist scores for quality scores, followed by Fischer post-hoc pairwise comparison (95%) test.

The descriptors generated by the panel to describe each quality category were grouped in categories according to semantic similarities by three experienced researchers using a triangulation task [27,28]. For each wine, the number of panellists who chose a category was counted. This citation frequency (%) was calculated as shown in Equation (1). Data analyses were carried out using XLSTAT (Addinsoft, version 2019).

(1) $$Citation\;frequency\;\left(\%\right)=\frac{Number\;of\;panellists\;who\;chose\;a\;term\;}{Number\;of\;total\;panellist}\times 100$$

### 2.3. Study of the Use of Resins to Remove Oxidation Aldehydes

#### 2.3.1. Wine Samples

To investigate the effect of sulphur dioxide on the removal of aldehydes, the same white wine (Verdejo variety) was studied at two different sulphur dioxide concentration levels: (1) wine native SO_2_ concentration, (2) white wine spiked with sodium metabisulphite to increase the free SO_2_ to levels around 40 mg/L.

#### 2.3.2. Resins

Three types of resins were used:

- Nucleophilic Scavengers (non-regenerable): Siliabond from SiliCycle (Quebec, QC, Canada) and Ethylenediamino purchased from Sigma-Aldrich.
- Anionic Mixed Mode (regenerable): Oasis MAX from Waters (Mildford, MA, USA), Bond Elut Certify II from Varian (Palo Alto, CA, USA), Strata X-A and Strata X-AW from Phenomenex (Torrance, CA, USA). This type of resins required a previous conditioning step with methanol and vacuum drying under nitrogen stream.
- Anionic Pure Mode (regenerable): Dowex^TM^ 1 × 2 50–100 and Amberlite^®^ IRA 900 were purchased from Sigma–Aldrich.

Resin characteristics are shown in Table 3.

#### 2.3.3. Experimental Procedure

Eight resins were tested in duplicate for two samples: a commercial white wine and the same wine with a higher concentration of SO_2_. The controls of the experiment were the wines with the two levels of SO_2_ incubated under the same conditions, but without resins.

The addition of resins was carried out inside an oxygen free chamber from Jacomex (Dagneux, France). Thirty-six 60 mL tightly screw capped clear glass vials supplied by WIT-France (Bordeaux, France), containing 10 g/L of the corresponding resin were used. The tubes were filled up completely with wine, and were carefully closed, avoiding any headspace. The tubes were incubated in a thermostatic bath Grant OLS23 with orbital shaking (130 rpm) at 25 °C. After 24 h, the tubes were introduced back into the anoxic chamber in order to decant the resins and then, the wines were analysed.

#### 2.3.4. Chemical Analysis

Oenological conventional parameters of colour, total polyphenol index (TPI), pH, total acidity, free sulphur dioxide, total sulphur dioxide, total acetaldehyde and total Strecker aldehydes were determined at time zero and after the treatment with resins.

Results were processed by means of analysis of variance (ANOVA), whereas mean values were compared by Fischer’s test (XLSTAT). The value of *p* ≤ 0.05 was considered statistically significant, and alphabetical letters were used along means in the tables.

##### Conventional Oenological Parameters

The total acidity was determined by acid-base volumetric titration measuring the end of the titration with a pH meter up to pH 7 according to the Organization of Vine and Wine (OIV) [29].

The pH was determined by potentiometry according to OIV practices [30].

For free and total sulphur dioxide determination, the aspiration/titration method recommended by the OIV was used [31].

##### Spectrophotometric Measurements

For colour determination, absorbances at wavelengths 420, 520 and 620 nm of undiluted wine samples were measured using glass cells with optical paths of 1, 2 or 5 mm, taking the measurement which provided absorbance readings between 0.3 and 0.7 as recommended by the OIV [32]. TPI was determined as absorbance at 280 nm as described by Ribéreau-Gayon et al. [33]. All the absorbance measurements were recorded using an UV−vis spectrophotometer UV-17000 Pharma Spec from Shimadzu (Kyoto, Japan).

##### Total Acetaldehyde Determination

Total acetaldehyde was determined by gas chromatography with flame ionization detection (GC-FID) by injection of 1 µL of wine sample spiked with 2-butanol as internal standard. The methodology is based on breaking the adducts directly in the injector port. A GC 8000 series from Fisons Instrument (Ipswich, UK) with a DB-WAX (30 m × 0.53 mm of i.d. × 2 μm) capillary column from J&W Scientific (Agilent Technologies, Santa Clara, CA, USA) were used. The injector was kept at 250 °C and the split ratio was 1:4. Hydrogen was used as carried gas and the pressure was kept at 27.5 kPa. The temperature program was 50 °C for 5 min and then raised to 220 °C in 10 min. The FID temperature was 250 °C. The calibration was obtained by the analysis of synthetic wines (5 g/L tartaric acid, 12% ethanol, 1.5% propane-1,2-diol, 10 g/L glycerin, pH 3.5) containing known amounts of acetaldehyde and plotting the corresponding peak areas (normalized by the internal standard) versus the mass of acetaldehyde. Other validation parameters are detailed elsewhere [24].

##### Total Strecker Aldehydes Determination

Total Strecker aldehydes content was determined as described by Bueno et al. [10]. In summary, vials were prepared inside an oxygen free chamber, pouring 10 mL of wine sample into a 20 mL vial. Then samples were spiked with methyl 2-methylbutyrate (187 µg/L) as internal standard and 6 g/L of glyoxal. Vials were then closed and incubated at 50 °C for 6 h for ensuring that carbonyl-bisulphite complexes had been broken. Total isobutyraldehyde, 2-methylbutanal, 3-methylbutanal, methional and phenylacetaldehyde were measured by headspace-solid phase microextraction followed by gas chromatography—mass spectrometry (HS-SPME-GC-MS) using a polydimethylsiloxane/divinylbenzene (PDMS/DVB) fibre from Supelco (Madrid, Spain).

##### Data Analysis

Results were analysed by analysis of variance (ANOVA), and for significant effects post-hoc Fischer’s test was calculated (XLSTAT). The value of *p* ≤ 0.05 was considered statistically significant, and alphabetical letters were used along means in the tables to indicate significant differences.

## 3. Results and Discussion

In the present work, the effect of the presence of Strecker aldehydes (released as a consequence of the depletion of sulphur dioxide) on the quality perceived by a group of Spanish technical experts in two different wine contexts has been evaluated. Further, the effect of different commercial resins on the elimination of these oxidation-related aldehydes has been tested.

### 3.1. Sensory Significance Derived from the Release of Strecker Aldehydes from Hydroxyalkylsulfonate Adducts

Strecker aldehydes in wine can be found in their free form, which is volatile and can be orthonasally perceived, or in their adduct form with SO_2_ (hydroxyalkylsulfonate), which is non-volatile and cannot be sensory perceived. In fact, it has been reported that non-oxidised commercial wines contain a pool of powerful oxidation-related Strecker aldehydes (from 33 to 96%) that are released back into their free volatile form when free SO_2_ disappears [8]. However, the concentration of Strecker aldehydes that are detrimental to wine aroma quality has not been studied yet.

**Figure 1:**
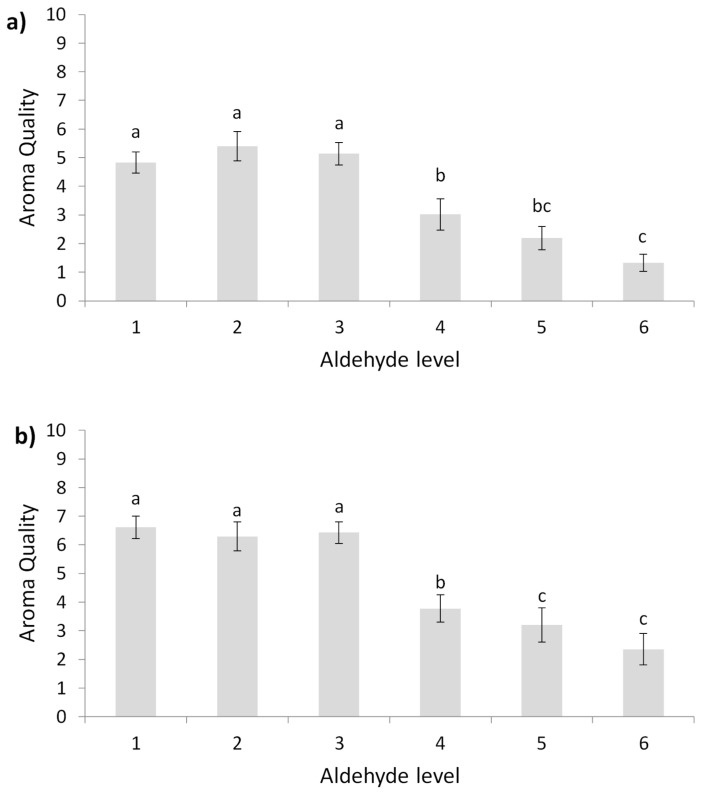
Average aroma quality for different levels of aldehydes. (**a**) Young wine model (**b**) Oaked wine model. Different letters within the same graphic indicate significant differences (*p* ≤ 0.05). Error bars are calculated as sd/n^0.5^; sd: standard deviation; *n*: number of panellists.

The quality scores given by the panel of experts for the six samples in each of the contexts indicates that the quality varies significantly (*p* < 0.001) with the level of aldehydes in both contexts. As can be seen in Figure 1, the increase in the concentration of aldehydes (from level 1 to level 6) causes a significant decrease in the perceived aroma quality in both contexts. In young wines, the release of Strecker aldehydes decreases orthonasal quality by between 27–40% and in oaked wines by 36–47% in both cases for the three aldehyde categories.

As can be seen in Figure 1, in both wine models, the maximum drop in quality is found from level 4. This implies that at the beginning of the shelf life of a wine (i.e., low levels of free aldehydes: levels 1–3), the initial concentration of free aldehydes regardless of the total amount of aldehydes (low, intermediate or high category) is at levels low enough to be hardly sensory perceived resulting in an absence of significant decrease in wine quality perception, according to the panel of wine experts. On the contrary, the ageing process (levels 4–6), with its implicit loss of SO_2_, causes the amount of free aldehydes to increase and thus to be sensory perceived resulting in a significant wine quality decrease.

Table 4 shows the decrease in quality scores due to the release of the free forms of aldehydes for similar theoretical levels of total aldehydes (e.g., high category: average quality score for the level 6 minus level 3 = −3.8 for the young wine). As it was expected, the panel of experts punishes the increment more in aldehyde concentration due to the simulation of the release of aldehydes from the breakdown of the aldehyde-SO_2_ adducts in wines with high amounts of total aldehydes (level 3 and level 6) compared to wines with low amounts (level 1 and level 4) (in both contexts). However, comparing the same aldehyde category in both wine models, quality depreciation was only significant for the lower aldehyde concentration (*p* ≤ 0.001), being more pronounced for oaked wines.

**Figure 2:**
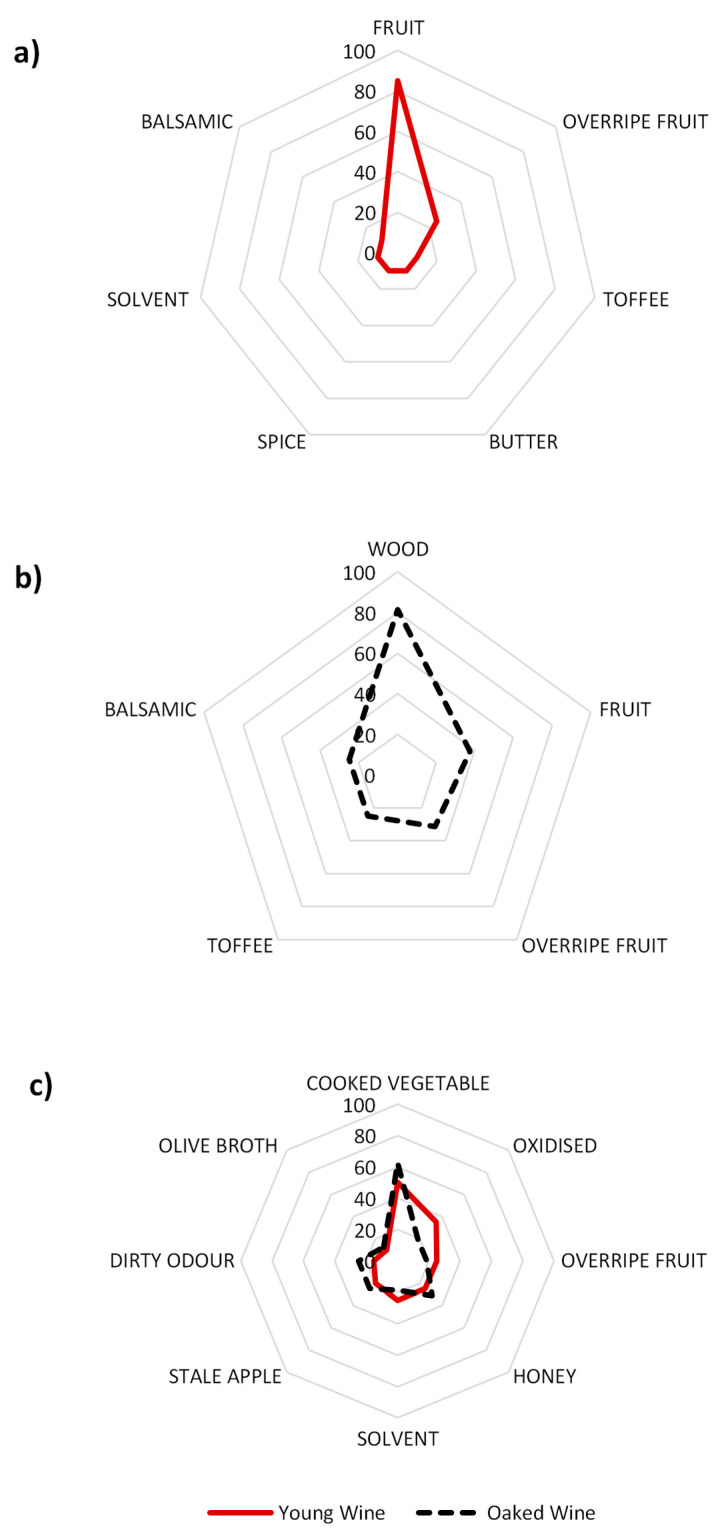
Citation frequency percentage of attributes related to (**a**) young wine with high aroma quality, (**b**) oaked wine with high aroma quality and (**c**) young and oaked wines with low aroma quality.

Figure 2 shows the spider graphs representing the citation frequencies of the attributes related to high quality or low quality in each wine context exemplars. In concordance with these frequencies, young wines with high quality would be mainly related to fruity aromas (Figure 2a), while low quality would be driven by descriptors such as cooked vegetables and oxidation-related attributes (Figure 2c).

This result suggests that the release of aldehydes during the oxidation of a young wine produces a masking effect on the fruity character of young wines, and the appearance of aromas related to cooked, oxidised vegetables, raisins, honey, alcoholic/fusel, stale apple and olive broth. For oaked wines, high quality seems to be represented by the presence of woody aromas (Figure 2b), while low quality wines would be related to a predominant note of cooked vegetables (Figure 2c). This seems to indicate that the increase in the concentration of aldehydes in their free forms produces a masking effect of the woody notes and the appearance of the negative attributes of aldehydes: cooked vegetables, honey, stale apple, dirty and oxidised.

It should be noted that the overripe fruit descriptor appears to be a descriptor linked to both high and low quality. This result suggests that there are two quality prototypes among the group of experts: 80% of them noted that this descriptor is a cue influencing positively wine quality against a 20% that find this descriptor to be involved in the detriment of wine quality. This is not an isolated example, Saenz-Navajas et al. observed that the same attribute could be related to high or low quality [34]. For high quality wine, this attribute can be tolerated by experts, whereas for low quality wines constitutes a rather negative feature.

Concerning the sensory results obtained, it is demonstrated that an appreciable sensory change occurs when the Strecker aldehydes from the hydroxyalkylsulfonates are released by depletion of SO_2_ due to the reversibility of this union, even without the need to form any de novo aldehyde molecule from its amino acid (Strecker reaction) or alcohol precursors (direct peroxidation). In addition, comparing with works previously published by our research group, it has been found that more than 75% of recently opened commercial wines contain total levels of Strecker aldehydes higher than those of level 4 (Table 2) [8,24].

### 3.2. Potential Ability of Different Commercial Resins to Eliminate Aldehydes Responsible for the Oxidation Notes in Wine

The sensory suppression of acetaldehyde by adding sulphur dioxide has been addressed in a previous work [35]. This SO_2_ addition binds acetaldehyde practically in a reversible way, due to the high adduct formation constant. However, although this SO_2_ addition solves the problem for this compound, it has been exposed in this paper that it would mask and delay a problem with Strecker’s aldehydes. Given the negative influence that all these aldehydes have on the orthonasal quality of wines at commonly found concentrations, it seems pertinent to study the potential capacity of different commercial resins to eliminate them, including acetaldehyde.

**Figure 3:**
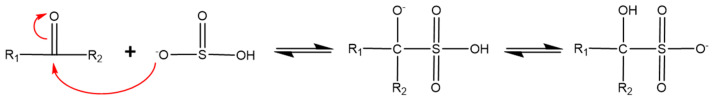
Hydroalkylsulfonate adduct formation equilibrium.

The removal evaluation has been carried out with a commercial white wine and the same wine spiked with potassium metabisulfite to test two different concentrations of SO_2_ in the wine. Two types of resins were tested: (1) nucleophilic scavengers that remove free aldehydes and force a shift of the SO_2_-aldehyde equilibrium (Figure 3), and (2) anion exchange that remove the hydroxyalkylsulfonate adducts. Within the latter type, pure anion exchange resins based on styrene–divinylbenzene with quaternary ammonium as a functional group and mixed mode anion exchange resins with different matrices and functional groups were used (see Table 3).

Table 5 shows the total concentrations of the six aldehydes in the control wines and the wines treated with each resin. The values provided by the quantitative methods are those of total aldehydes, for this reason and despite the fact that SO_2_ content is different, the values obtained for both controls are the same (there are no significant differences).

In general, the reproducibility of the elimination process was satisfactory. Most of the relative standard deviations (RSD) were less than 15%, with the exception of SLB resin for the removal of the more volatile aldehydes (isobutyraldehyde, 2-methylbutanal and 3-methylbutanal) at both levels of SO_2_.

The removal percentages obtained were relevant. For isobutyraldehyde, they reached 22%, except for SXAW resin where a significant increase (*p* = 0.03) was observed at higher levels of SO_2_. For 2-methylbutanal, the decrease ranged from 0.2 to 47%. Nearly all the resins showed significant reductions in their amounts of 3-methylbutanal and methional compared to the control for both SO_2_ levels (Table 5) achieving elimination percentages up to 46% and 43%, respectively.

Phenylacetaldehyde increased its concentration after 24 h in contact with the scavengers. This type of resin cannot remove phenylacetaldehyde at all, and at the end of the process the concentration of aldehyde has increased. Two possible options are considered to explain this fact:

(a) Some nucleophile that is present in the resin reaches to break stable adducts (adducts that the method of determination of total aldehydes using glyoxal is not able to break).

(b) Another possibility will be that some trace contaminant, such as the amino acid phenylalanine, has been transferred from the resin and if there are α-dicarbonyls present in the medium, the Strecker reaction has occurred.

Notwithstanding, phenylacetaldehyde showed good elimination results using mixed mode resins such as MAX, SXA and SXAW (Table 5). These resins achieved removal percentages higher than 68% for the two sulphur dioxide levels. These types of resins have aromatic rings in their structures that would stabilize with the phenylacetaldehyde reaction. In addition, they have a large pore size (73–98 A) and specific surface area (727–889 m^2^/g), making the reaction with phenylacetaldehyde, which is a voluminous molecule, more favourable. However, pure anion exchange resins exhibit less removal for this compound.

Acetaldehyde exhibited removals up to 20% with DOW pure anion exchange resin. These removal percentages are very high if we compare with previous works where the concentration of total acetaldehyde did not change significantly, when an ionic exchange in column was applied to a wine of the Airén variety in order to improve its colour [36].

Briefly, the resins that are repeated more times with significant differences, with respect to the control for all aldehydes (Table 5), are principally DOW and SXA for both SO_2_ levels.

Furthermore, by increasing the SO_2_ level, the formation of hydroxyalkylsulfonates would be favoured and, in principle, a greater reducing effect of the pure and mixed mode ion exchange resins would be expected. Nevertheless, data show that only the removal of acetaldehyde has been significantly favoured with the pure ion exchange resins DOW (*p* = 0.017) and IRA (*p* = 0.018). In the case of phenylacetaldehyde, a greater reduction was also found due to the effect of sulphur dioxide in the DOW resin (*p* = 0.05).

The existence of significant effects only for these two aldehydes may be due to the fact that acetaldehyde is the most reactive compound with SO_2_ [9], and although the formation constant of the adduct with methional is greater than that of phenylacetaldehyde (50 × 10^3^ versus 17 × 10^3^ [10]), the phenylacetaldehyde adduct could be stabilized on the aromatic ring structures of mixed resins.

### 3.3. Effects of Resin Treatment on Other Wine Parameters

To assess other effects produced by the treatments with resins, free SO_2_, total SO_2_, pH, total acidity, TPI and colour of all the samples were analysed before and after the treatment (Table 6).

For the SLB scavenger, a higher concentration of free sulphur dioxide (16%) is obtained after incubation, showing that the process has worked properly, and the equilibrium has been displaced. In the test with the same wine spiked with SO_2_, only a 3% increase of free SO_2_ was found, which may indicate that the breakdown of the hydroxyalkylsulfonate adducts due to the removal of the aldehyde is not favourable at high adduct concentrations (see Table 6). The other scavenger, ETDM, seems to have been less effective if this parameter is taken into account, however, almost the same difference is again observed between the wine with the native content of SO_2_ or the one with higher amount.

Regarding ion exchange resins, the ones that react directly with the adduct, it was expected that the free and total sulphur dioxide would decrease as the process progressed. Indeed, this is effectively what has been obtained and is reflected in Table 6. Having an initial wine with a higher sulphur dioxide content increases the percentage of SO_2_ removal due to the treatment, especially with the pure ion exchange resins DOW and IRA.

Regarding TPI and colour, the resins behaved in the same way regardless of the level of SO_2_ in the initial wine. The decrease in these parameters may mainly be due to the elimination of catechins and hydroxycinnamic acids [36]. Hermonsín et al. established in their work the selection of ion exchange resins, a maximum colour elimination criterion of 35% in order not to compromise the rest of the sensory properties [36]. As can be seen in Table 6, only the scavengers would fulfil this premise, obtaining a marked decrease in colour (higher than 63%) for the rest of the resins, except for BEC.

Regarding the pH and total acidity, only the treatment with ETDM and SXAW resins provided a notable variation. In these two cases, the pH increased and the total acidity decreased, which is totally undesirable at an oenological level, since it compromises the microbial stability of the wine.

It must be considered that the concentration of phenylacetaldehyde increased after the treatment with scavenger resins. Moreover, one of the two resins of this type sharply decreased the total acidity of the wine. Therefore, the use of scavengers in this context can be ruled out. Thus, reaching a compromise, it can be concluded that the resins with the best behaviour are the mixed ion exchange SXA, both for the elimination of aldehydes, except isobutyraldehyde, and for maintaining the pH and total acidity to a great extent, closely followed by MAX resin. However, the colour decrease is greater than 75% in both cases. Perhaps by using a lower dose than the one studied in this work (10 g/L), the process would be more viable from a sensory and economic point of view.

## 4. Conclusions

This work has revealed that in the context of young wine and oaked wine, the release of aldehydes due to the breakdown of the aldehyde-SO_2_ adducts causes a clear depreciation of the orthonasal aroma quality in wines. Furthermore, this depreciation is more evident when the total aldehyde levels are high. From a descriptive point of view, the fruity character of young wines is masked by showing attributes that define oxidation. Quality detriment begins at a tested level 4, which contains the following aldehyde concentrations: 13 μg/L of methional, 49 μg/L of phenylacetaldehyde, 17 μg/L of isobutyraldehyde, 12 μg/L of 2-methylbutanal and 24 μg/L of 3-methylbutanal. More than 75% of recently opened commercial wines contain concentrations of total aldehydes higher than these, therefore, it seems reasonable to look for tools that reduce the presence of aldehydes responsible for oxidation aromas in wines.

Working with white wine, mixed ion exchange resins have offered the best aldehyde removal results, maintaining, predominantly, the pH and total acidity, although reducing the colour by at least 20%. On the other hand, the addition of sulphur dioxide only significantly improves the removal of acetaldehyde and phenylacetaldehyde using pure ion exchange resins.

Finally, the resin that has offered the best results has been the Strata X-A resin, which provides around 11–17% removal for isobutyraldehyde, 31–47% for 2-methylbutanal, 29–31% for 3-methylbutanal, 33–37% for methional, 78–86% for phenylacetaldehyde and 3–10% for acetaldehyde. However, due to the dose of resin used in the experiment (10 g/L), further investigation of this issue on an industrial scale would be necessary in order to reach applicability conclusions.

## Figures and Tables

**Table 1:** Wine models composition.

Compounds	Concentration (mg/L)	
	Young Wine	Oaked Wine
Isoamyl alcohol	180	
β-Phenylethanol	30	
Acetic acid	150	
Ethyl acetate	50	
Hexanoic acid	2.0	
3-Methylbutanoic acid	0.30	
2,3-Butanedione	0.40	
Ethyl hexanoate	1.0	
Isoamyl acetate	1.0	
Ethyl 2- methylbutanoate	0.12	
Ethyl vanillate	0.55	
β-Damascenone	0.0040	
β-Ionone	0.00030	
γ-Nonalactone	0.020	
Guaiacol	0.010	0.030
Vanillin	0.070	0.17
Whisky lactone	0	0.20
Eugenol	0	0.020
4-Hydroxy-2,5-dimethyl-3(2 H)- furanone	0	0.10
Acetovanillone	0	0.20
pH	3.5	
Glicerine (g/L)	10	
Quinine (mg/L)	7.0	
Arabic gum (mg/L)	75	
Ethanol (%)	12	
Tannic acid (mg/L)	50	100
Tartaric acid (g/L)	5.0	4.0

**Table 2:** Strecker aldehydes concentrations (μg/L) in wine models without sulphur dioxide.

	Level 1	Level 2	Level 3	Level 4	Level 5	Level 6
Isobutyraldehyde	4.30	8.50	14.2	16.6	33.2	55.3
2-Methylbutanal	3.70	7.30	12.2	11.7	23.4	38.9
3-Methylbutanal	2.50	5.00	8.50	24.3	48.6	82.6
Methional	1.30	2.40	4.00	13.5	25.6	43.1
Phenylacetaldehyde	4.70	8.50	14.4	49.2	88.6	149

**Table 3:** Characteristics of the resins considered in the study.

Type	Name	Code	Type Specification	Matrix	Functional Group	Particle Size (μm)	Pore Size (A)	Surface Area (m^2^/g)	Capacity
Nucleophilic Scavengers	Siliabond	SLB	Scavenger	Silica		40–63	55–65	480–550	0.70 mmol/g
	Ethylendiamino	ETDM	Scavenger	Silica			60	500	1.40 mmol/g
Anionic Mixed Mode	Oasis MAX	MAX	Polymeric + SAX	PVP-DVB	DMB-QA	60	73–98	727–889	0.25 meq/g
	Bond Elut Certify II	BEC	Polymeric + SAX	C8	TM-QA	40–120	60	500	0.85 meq/g
	Strata X-A	SXA	Polymeric + SAX	PS-DVB	DMB-QA	33	85	800	0.30 meq/g
	Strata X-AW	SXAW	Polymeric + WAX	PS-DVB	1,2-DA	33	85	800	0.60 meq/g
Anionic Pure Mode	Dowex 1×2 50–100	DOW	SAX	Styrene -DVB	QA	73–149	-	-	0.7 meq/mL
	Amberlite IRA-900	IRA	SAX	Styrene -DVB	QA	650–820	-	-	1 meq/mL

SAX: strong anion exchange; WAX: weak anion exchange; PVP: polyvinylpyrrolidone; DVB: divinylbenzene; C8: octyl group; PS: polystyrene; DMB: dimethyl butyl; QA: quaternary ammonium; TM: trimethyl; 1,2-DA: primary and secondary diamino.

**Table 4:** Aroma quality differences due to the release of bound aldehydes.

Differences Due to the Release of Bound Aldehydes	Young Wine	Oaked Wine	*p*-Value (Context)
High category (level 6–level 3)	−3.8 ^a^	−4.1 ^a^	0.077
Intermediate category (level 5–level 2)	−3.2 ^b^	−3.1 ^b^	0.206
Low category (level 4–level 1)	−1.8 ^cB^	−2.8 ^bA^	<0.001
*p*-value (category)	<0.001	<0.001	

a–c: different superscripts within the same column denote statistical differences (*p* ≤ 0.05) between different aldehyde category for the same wine context. A–B: different superscripts in the same row denote statistical differences (*p* ≤ 0.05) between wine context for the same aldehyde category.

**Table 5:** Total Strecker aldehydes (μg/L) and acetaldehyde (mg/L) concentrations after 24 h at 25 °C in contact with the resins.

Levels of SO_2_		Isobutyraldehyde		2-Methylbutanal		3-Methylbutanal		Methional		Phenylacetaldehyde		Acetaldehyde	
		Mean	RSD (%)	Mean	RSD (%)	Mean	RSD (%)	Mean	RSD (%)	Mean	RSD (%)	Mean	RSD (%)
Free SO_2_ 12.8 mg/L Total SO_2_ 142.8 mg/L	Control	10.6 ^ab^	6.2	4.85 ^a^	9.4	50.5 ^a^	4.2	9.80 ^a^	5.5	31.6 ^bc^	1.2	49.7 ^ab^	1.8
	SLB	5.40 ^d^	46	1.22 ^e^	57	33.9 ^ef^	14	7.11 ^bcd^	15	415 ^a^	1.6	46.0 ^bc^	11
	ETDM	9.80 ^bc^	4.7	3.97 ^ab^	0.7	49.5 ^ab^	1.8	10.1 ^a^	4.7	35.9 ^b^	2.7	51.5 ^a^	2.3
	MAX	9.40 ^bc^	7.6	3.68 ^bc^	7.9	35.5 ^def^	12	5.65 ^e^	18	<LQ^e^	_	47.7 ^a^	0.1
	BEC	9.50 ^bc^	1.9	4.00 ^ab^	8.1	48.0 ^ab^	2.0	7.87 ^bc^	1.5	21.1 ^d^	14	51.0 ^abc^	2.6
	SXA	8.80 ^bc^	11	2.53 ^d^	14	35.8 ^de^	7.6	6.19 ^de^	8.3	<LQ^e^	_	44.8 ^cd^	3.1
	SXAW	12.5 ^a^	8.8	3.15 ^bcd^	22	30.1 ^f^	3.0	6.79 ^cde^	0.9	<LQ^e^	_	43.5 ^cd^	0.9
	DOW	8.30 ^c^	4.1	2.91 ^cd^	5.7	40.0 ^cd^	0.1	6.83 ^cde^	3.7	25.5 ^cd^	2.4	41.0 ^d^	1.0
	IRA	8.90 ^bc^	0.6	3.44 ^bc^	6.0	44.6 ^bc^	3.8	8.38 ^b^	5.0	25.8 ^cd^	5.7	44.0 ^cd^	1.1
*p*-value (native SO_2_)		0.005		<0.001		<0.001		<0.001		<0.001		0.004	
Free SO_2_ 36.8 mg/L Total SO_2_ 172.8 mg/L	Control	10.0 ^BC^	2.3	4.71 ^A^	14	47.5 ^A^	2.2	9.21 ^B^	1.7	28.5 ^C^	0.5	48.5 ^A^	6.5
	SLB	4.80 ^D^	50	1.31 ^C^	57	34.8 ^C^	26	7.60 ^C^	6.0	363 ^A^	1.5	40.1 ^C^	8.3
	ETDM	10.8 ^AB^	0.8	4.74 ^A^	0.4	47.2 ^AB^	1.4	11.2 ^A^	7.9	41.6 ^B^	3.1	47.5 ^A^	3.6
	MAX	9.30 ^BC^	4.5	4.67 ^A^	1.5	39.4 ^BC^	4.0	6.40 ^CD^	2.3	< LQ ^E^	_	45.7 ^A^	0.3
	BEC	10.1 ^ABC^	1.3	4.63 ^A^	12	47.1 ^AB^	1.1	7.50 ^C^	1.5	22.9 ^CD^	3.1	45.3 ^A^	1.7
	SXA	8.90 ^BC^	8.6	3.32 ^B^	8.8	32.8 ^CD^	11	6.10 ^D^	23	<LQ ^EF^	_	46.9 ^A^	2.9
	SXAW	12.3 ^A^	4.3	3.80 ^AB^	0.1	25.5 ^D^	5.4	5.90 ^D^	7.7	<LD ^F^	_	44.4 ^AB^	3.9
	DOW	8.50 ^C^	4.7	3.91 ^AB^	4.2	36.7 ^C^	1.7	6.90 ^CD^	2.4	19.6 ^D^	15	38.8 ^C^	1.2
	IRA	9.80 ^BC^	13	4.67 ^A^	6.9	40.4 ^ABC^	7.6	7.00 ^CD^	4.7	19.2 ^D^	19	40.6 ^BC^	2.0
*p*-value (high SO_2_ level)		0.002		<0.001		0.002		<0.001		<0.001		0.003	

RSD: relative standard deviation; LD: detection limit of phenylacetaldehyde (3 μg/L); LQ: quantification limit of phenylacetaldehyde (10 μg/L). a–f: different superscripts within the same column denote statistical differences (*p* ≤ 0.05) between different resin treatments for wine with native SO_2_ content. A–F: different superscripts within the same column denote statistical differences (*p* ≤ 0.05) between different resin treatments for wine with high SO_2_ level.

**Table 6:** Removal percentage of SO_2_, colour and total polyphenol index (TPI) after 24 h of treatment with resins. Total acidity, TA (g/L tartaric acid) and pH after 24 h.

	Removal Percentage								Final Data			
	SO_2_ Free		SO_2_ Total		TPI		Colour		TA		pH	
	T1	T2	T1	T2	T1	T2	T1	T2	T1	T2	T1	T2
Control									5.4	5.4	3.2	3.1
SLB	−16	−3.5	9.0	31	0.14	−4.7	29	32	5.3	5.4	3.2	3.2
ETDM	0.10	13	17	23	12	10	16	20	3.5	3.6	3.7	3.8
MAX	11	37	22	27	45	42	76	75	5.3	5.4	3.2	3.1
BEC	6.0	11	27	16	8.6	3.8	24	20	5.6	5.6	3.2	3.1
SXA	13	19	38	34	48	44	85	83	5.2	5.3	3.2	3.1
SXAW	0.40	18	34	29	31	23	72	75	4.5	4.6	3.6	3.4
DOW	0.30	30	45	52	39	38	68	68	5.1	5.1	3.1	3.0
IRA	13	35	28	57	44	45	63	64	5.2	5.1	3.0	3.0

T1: test with the wine initial SO_2_ level (Free SO_2_: 12.8 mg/L. Total SO_2_: 142.8 mg/L). T2: test with wine spiked with SO_2_ (Free SO_2_: 36.8 mg/L. Total SO_2_: 172.8 mg/L).
